# Desialylation of Sonic-Hedgehog by Neu2 Inhibits Its Association with Patched1 Reducing Stemness-Like Properties in Pancreatic Cancer Sphere-forming Cells

**DOI:** 10.3390/cells9061512

**Published:** 2020-06-21

**Authors:** Shalini Nath, Susmita Mondal, Ramesh Butti, Vinoth Prasanna Gunasekaran, Uttara Chatterjee, Aniket Halder, Gopal C. Kundu, Chitra Mandal

**Affiliations:** 1 Cancer Biology and Inflammatory Disorder Division, Council of Scientific and Industrial Research-Indian Institute of Chemical Biology, 4, Raja S.C. Mallick Road, Kolkata 700032, India; shalininath@csiriicb.res.in (S.N.); susmita.blysci@gmail.com (S.M.); 2 Laboratory of Tumor Biology, Angiogenesis and Nanomedicine Research, National Centre for Cell Science (NCCS), Pune 411007, India; rameshbutti@gmail.com (R.B.); vinothprasannagm@gmail.com (V.P.G.); gopalc.kundu@kiit.ac.in (G.C.K.); 3 Department of Pathology, Institute of Post-Graduate Medical Education and Research Hospital, Kolkata, West Bengal 700020, India; uttarac1@gmail.com; 4 School of Digestive & Liver Diseases, Institute of Post-Graduate Medical Education and Research Hospital, Kolkata, West Bengal 700020, India; aniket.halder@gmail.com

**Keywords:** apoptosis, desialylation, mTORC2, Neu2, pancreatic cancer sphere-forming cells, sialidase, sialic acids, Sonic hedgehog pathway, sialylation, stemness-like property

## Abstract

Cancer stem cells (CSCs) are crucial regulators of tumor recurrence/progression. The maintenance of CSCs is dependent on aberrant activation of various pathways, including Hedgehog. Prevalent sialylations contribute to aggressiveness in CSCs. Here, we have addressed the role of sialylation in regulating stemness-like properties of pancreatic cancer sphere-forming cells (PCS) through modulation of the Hedgehog (Hh) pathway. The status of CD133/CD44/surface-sialylation was checked by flow cytometry and effects of Neu2 overexpression in PCS were compared using qPCR, immunoblotting, co-immunoprecipitation and also by colony-formation assays. The work was also validated in a xenograft model after Neu2 overexpression. Neu2 and Shh status in patient tissues were examined by immunohistochemistry. PCS showed higher Hh-pathway activity and sialylation with reduced cytosolic-sialidase (Neu2). Neu2 overexpression caused desialylation of Shh, thereby reducing Shh-Patched1 binding thus causing decreased Hh-pathway activity with lower expression of Snail/Slug/CyclinD1 leading to reduction of stemness-like properties. Neu2-overexpression also induced apoptosis in PCS. Additionally, Neu2-overexpressed PCS demonstrated lower mTORC2 formation and inhibitory-phosphorylation of Gsk3β, reflecting a close relationship with reduced Hh pathway. Moreover, both Neu2 and Rictor (a major component of mTORC2) co-transfection reduced stem cell markers and Hh-pathway activity in PCS. Neu2-overexpressed tumors showed reduction in tumor mass with downregulation of stem cell markers/Shh/mTOR and upregulation of Bax/Caspase8/Caspase3. Thus, we established that reduced sialylation by Neu2 overexpression leads to decreased stemness-like properties by desialylation of Shh, which impaired its association with Patched1 thereby inhibiting the Hh pathway. All these may be responsible for enhanced apoptosis in Neu2-overexpressed PCS.

## 1. Introduction

Cancer stem cells (CSCs) are a population of cancer cells having self-renewal and multipotency abilities [1,2]. CSCs demonstrate an uncontrolled Hedgehog (Hh) signaling pathway which drives tumor progression [3,4]. Constitutive activation of these pathways in CSCs leads to tumor cell survival [5,6]. In general, the Hedgehog-pathway is activated when Shh binds with Patched1 (PTCH1). Such binding helps to release a Smo molecule which further leads to activation of transcription factor Gli [7,8]. Additionally, mTORC2 signaling also facilitates Hh-pathway activity by promoting nuclear translocation of Gli proteins through inhibitory phosphorylation of the GSK3β molecule in glioblastoma cell lines [9]. 

The status of sialylation is controlled mainly by two groups of enzymes, namely sialidases and sialyltransferases, which cleave and add sialic acids, respectively, to carbohydrate chains of glycosylated molecules [10,11,12,13,14]. Hence, the altered expressions of these enzymes can affect various biological phenomena like cellular signaling, invasiveness, metastasis and apoptosis during carcinogenesis [15]. The four mammalian sialidases, namely Neu1 (lysosomal), Neu2 (cytosolic), Neu3 (membrane-bound) and Neu4 (lumen of lysosomes, endoplasmic reticulum and mitochondria) present in the mammalian cells act differently during carcinogenesis [15]. The expression of Neu2 is low in many cancers like colon adenocarcinoma and leukemia [16,17]. Recently, we have reported reduced expression of Neu2 in pancreatic cancer [18], the fourth leading source of cancer-related deaths worldwide [19,20,21]. Glycoproteins, glycolipids and oligosaccharides with both α2,3- and α2,6-linked sialic acids are the substrates of Neu2 [15]. However, the role of Neu2 in pancreatic cancer stem cells predominantly driven by the Hh-signaling pathway has received the least attention. Additionally, the involvement of mTORC2 which sequentially controls the aberrant Hh-pathway regulation has also not been addressed in the context of Neu2 in pancreatic CSCs. 

Initially, we generated spheres from pancreatic cancer cell lines showing higher CSC markers, higher sialylation status and upregulated Hh-pathway molecules. Patient tissues exhibited lower Neu2 as well as higher Shh than their normal counterpart, the same trends which have been followed in pancreatic cancer sphere-forming cells (PCS). Accordingly, we aim to establish the role of Neu2 on modulation of the Hh pathway in PCS. Here, we demonstrated that overexpression of Neu2 caused its enhanced binding with Shh, causing desialylation of Shh. As a result, there was a decreased association of Shh with Patched1, leading to inhibition of several Hh-pathway molecules. 

Additionally, Neu2-overexpressed PCS demonstrated reduced mTORC2 formation and decreased Hh-pathway molecules and target genes by downregulating inhibitory phosphorylation of Gsk3β. However, overexpression of Rictor leads to improved stemness-like properties and higher Hh activity. Interestingly, co-expression of Rictor and Neu2 reverted this consequence, indicating modulation of PCS characteristics both via mTORC2 signaling and Shh desialylation through Neu2. Furthermore, this was corroborated by an in vivo study where Neu2 overexpression caused reduction of tumor size and volume in NOD/SCID mice. These tumor tissues showed downregulation of several stem cell markers, Shh and mTOR proteins as well as upregulation of pro-apoptotic (BAX/CAS3/CAS8) genes. 

Taken together, for the first time, we have demonstrated that Shh is a sialoglycoprotein with both α2,3- and α2,6-linked sialic acids. Therefore, an enhanced association of Neu2 with Shh plays an important role in reducing the stemness-like properties of PCS. Here, we have also revealed the underneath molecular cross-talk between Neu2/mTORC2/Hh pathway in PCS.

## 2. Material and Methods

### 2.1. Chemicals

Iscove’s Modified Dulbecco’s Medium (IMDM), fetal bovine serum (FBS), Lipofectamine LTX, plus reagent, matrigel, DMEM/F12, B27 supplement, N2 supplement, and Anti-Neu2 were from Invitrogen (MA5-25555) (USA), as well as CD133 and CD44 (BD phermingen) and Oct-4A(2840), Sox2(3579), Nanog(4903), Slug(9585S), Snail(3879S), Cyclin D1(2978S), mTOR(2983), phospho-mTOR (Ser2481)(2974), Rictor(9476), GSK-3β(9315), Gli1(3538S), Sufu(2522S),GSK-3β(9315), Phospho-GSK-3β-Ser9(5558), Akt(4691), Phospho-Akt-Ser473(4060), β-Actin(4970), HRP-conjugated anti-rabbit antibodies(7047S) and anti-mouse secondary antibodies (7076). SignalStain^®^ DAB Substrate Kit(8059) was from Cell Signaling Technology (Danvers, USA). Shh(sc-9024) antibody was from Santa Cruz Biotechnology (Texas, USA). Basic fibroblast growth factor (bFGF), epidermal growth factor (EGF), anti-Caspase3, active antibody(C8487) and other chemicals were from Sigma–Aldrich. RevertAid First Strand cDNA Synthesis Kit (K1622) and Maxima SYBR Green qPCR Master Mix (2X) were from Thermo Scientific, USA (K0251); biotinylated Sambucus nigra agglutinin (SNA)(B-1305) and biotinylated Maackia amurensis agglutinin (MALII) (B-1265) were from Vector Laboratories (USA).Rictor plasmid Addgene plasmid #11367) was from Addgene and PcDNA3.1-Neu2 was a kind gift from Dr. Eugenio Monti. 

### 2.2. Cell Culture

MIAPaCa2 and PANC1 were cultured in IMDM medium, AsPC1, and BxPC3 cells were cultured in RPMI-1640 medium, supplemented with FCS (10%), L-glutamine (0.002 M), antibiotics, and antimycotics. Pancreatic cancer sphere-forming cells (PCS) were generated from all four cell lines by culturing them in a stem cell-specific medium containing DMEM/F12, B27 supplement, EGF and bFGF in ultra-low attachment plates for 72 h.

### 2.3. Flow Cytometry

Adherent pancreatic cancer cells and PCS cells (5 × 10^5^) from both MIAPaCa2 and AsPC1 cells were collected, washed and resuspended in phosphate buffered saline (PBS, 0.02M, pH 7.2, 100 µL), and incubated with anti-CD133-APC and CD44-PE antibodies (BD PharMingen) for 30 min at 4 °C in the dark [3]. Subsequently, data were acquired using flow cytometry (FACS LSRFortessa) and were analyzed using FACSDiva 8.0.2 software. 

### 2.4. Real-Time PCR analysis

Total RNA from cells was extracted using a RNeasy mini kit following the manufacturer’s instructions. First strand cDNA was synthesized by RevertAid First Strand cDNA Synthesis Kit from Thermo Scientific (USA) according to the manufacturer’s protocol [21]. Real-time PCR was performed using a Maxima SYBR Green qPCR Master Mix from Thermo Scientific (USA). LightCycler 96 (Roche, Basel, Switzerland) software was used to quantify relative amounts of target mRNA with 18S rRNA as an internal control. A primer list is provided in Supplementary Table S1. 

### 2.5. Immunoblotting

Adherent pancreatic cancer cells, PCS, Neu2-overexpressed PCS (N-PCS), Rictor overexpressed PCS and Neu2+Rictor overexpressed PCS were harvested separately. Cells were lysed and processed for Western blotting using appropriate antibodies [18,22]. Images of the developed bands were captured by BioradChemiDoc MP System (Bio-Rad, Hercules, CA, USA). Some of the blots were developed by Azure c400 Visible Fluorescent Western Blot Imaging System.

### 2.6. Glycoprotein Analysis

Cell lysate of PCS and adherent pancreatic cancer cells, along with Neu2-overexpressed PCS (N-PCS), were separated by 10% SDS-PAGE. Biotinylated SNA and MALII were used for detection of α2,6- and α2,3-linked sialioglycoproteins. Blots were developed with avidin-HRP. Ponceau S-stained blots indicate equal loading.

### 2.7. Detection of Linkage-Specific Sialic Acids 

The presence of linkage-specific sialic acids was investigated by using SNA and MALII. Adherent pancreatic cancer cells and PCS (1 × 10^5^) cells were washed and suspended in lectin-binding buffer (20 mM Tris, 0.5 M NaCl, 2.0 mM MnCl_2_, 2.0 mM MgCl_2_, 2.0 mM CaCl_2_) [18,23]. Fluorescein isothiocyanate (FITC)-conjugated SNA and MALII were incubated separately with cells for 1 h (5.0 μg/mL) at 4 °C and acquisition of FITC positivity was done by Flow cytomerty. 

### 2.8. Transfection 

Pancreatic cancer sphere-forming cells (PCS) were initially generated from MIAPaCa2 and AsPC1 cells in stem cell-specific medium at 90% confluence. The spheres collected from the supernatant were dispersed, washed and taken in stem cell-specific medium without growth factors in a 6-well plate (1 × 10^6^/well). They were subsequently transfected with PcDNA3.1-Neu2 vector for 24 h using lipofectamine LTX. The transfection mixture was removed the following day and replaced with stem cell-specific medium up to 48 h [3]. These cells were also transfected with pRK-5/Rictor vector alone as well as co-transfected with both pRK-5/Rictor and PcDNA3.1-Neu2 vectors for 24 h and processed similarly. 

### 2.9. Sphere Formation Assays

Neu2-transfected PCS and PCS (1 × 10^5^) were trypsinized and further seeded for sphere formation in stem cell-specific medium for 48 h. Images of spheres were taken by using phase-contrast microscopy (EVOS, Life Technologies) [3].

### 2.10. Confocal Microscopy 

Adherent pancreatic cancer cells, PCS and Neu2-transfected PCS (1 × 10^4^/well) were plated on poly-L-Lycine coated coverslips for 3 h, washed with PBS-0.3% Tween 20 (PBS-T) and fixed with paraformaldehyde. Cells were initially incubated with goat serum for 1.0 h at 25 °C and then with primary antibodies overnight at 4 °C followed by incubation with Alexa Fluor 594-conjugated secondary antibodies for 3.0 h at 25 °C [3]. Cells were visualized after washing with PBS-T using the Zeiss inverted confocal microscope. 

### 2.11. Cell Viability Assay

PCS and N-PCS (1 × 10^4^) were seeded to 96-well ultra-low attachment plate for 48 h and consequently, 3-(4,5-dimethylthiazol-2-yl)-2,5-diphenyl tetrazolium bromide (MTT, 100 μg per well) was added to culture media and further incubated for 3 h. Plate was centrifuged and generated formazan crystals were dissolved in DMSO and optical density was measured at 550 nm in a spectrophotometer (Thermo Scientific, USA).

### 2.12. Patient Samples

The immunohistological study with human patient tissues; tumor (n = 10) and adjacent non-tumor (n = 10) specimens, had been performed on the archived samples from GI pathology department, of Institute of Post-Graduate Medical Education and Research Hospital at Kolkata, India, after proper informed consent from each patients. The study was performed strictly according to the guidelines framed by Indian Council of Medical Research, 2017, in accordance with research protocol. The reference number of the approval permission of ethical committee is IPGME&R/IEC/2019/198, dated 12 April 2019. 

### 2.13. Immunohistochemistry

The pancreatic and normal tissues were used for immunohistological staining after sectioning by microtome [18]. For the recovery of antigen, the sections were heated in 0.01 M citrate buffer (pH 6.0) and incubated with the anti-Neu2 and anti-Shh antibodies overnight. Horseradish peroxidase conjugated anti-mouse and anti-rabbit antibodies were used as secondary antibodies, respectively. Then, these tissue sections were developed with SignalStain^®^ DAB Substrate Kit. These samples were photographed at ×20 magnification. ImageJ software was used to measure IHC optical density score. Neu2 and Shh were detected in 10 different patient tissues. Adjacent normal tissues were processed similarly and used as controls for comparison.

### 2.14. Co-Immunoprecipitation

The association of Shh with Neu2 and Shh with Patched1 was detected both in PCS and N-PCS. These cells were lysed by sonication (Qsonica-LLC, XL-2000 series, Newtown, CT, USA) and protein was collected after centrifugation at 800× *g* for 10 min [24]. The proteins (200 μg) from cell lysate were incubated with the anti-Shh and anti-Patched1antibody (1:100) separately overnight at 4 °C. The immuno-complex was incubated with protein A-Sepharose 4B for 3 h. Beads were washed with PBS and incubated with sample buffer without β-ME. The proteins were separated by SDS-PAGE and subsequently identified using anti-Neu2, anti-Patched1 and anti-Shh antibodies separately. 

Similarly, to detect the status of α2,6- and α2,3-linked sialic acids on Shh, cell lysate from N-PCS was initially incubated with the anti-Shh antibody and immunocomplexes were resolved by SDS-PAGE. These were subsequently detected by biotinylated SNA and MALII and then developed with avidin-HRP. PCS cells were processed similarly for comparison.

### 2.15. In Vivo Tumorigenicity

The animal studies were performed in compliance with the guidelines of the Institutional Animal Care and Use Committee (IACUC) of the National Centre for Cell Science, Pune, India. Briefly, MIAPaCa2 (1 × 10^7^) cells were injected subcutaneously into the dorsal side of the right flanks of 6-week-old male NOD/SCID mice to develop xenograft tumors. After 21 days, we observed detectable tumors and mice were randomly divided into two groups. One group was injected with vehicle control whereas the other group was injected intratumorally with 1.5 mg/kg body wt. PcDNA3.1-Neu2 plasmid in admixture with Lipofectamine 2000 (1:2) twice a week for 3 weeks [25,26,27]. Tumor size was monitored periodically. Mice were sacrificed after 30 days and tumor size and volume were measured.

### 2.16. Statistical Analysis

All these data collected from three independent experiments and statistical analysis was performed using Graph Pad Prism 5. Two tail Student’s *t*-test was used to detect the differences between the groups. Standard error bars represent the standard deviation of the mean (±SD) and *p*-values (* *p* < 0.05; ** *p* < 0.01; *** *p* < 0.001) represented the significant differences between the means of the two test groups. 

## 3. Results 

### 3.1. Generation and Characterization of Pancreatic Cancer Sphere-Forming Cells (PCS) from an Array of Pancreatic Cancer Cell Lines

**Figure 1:**
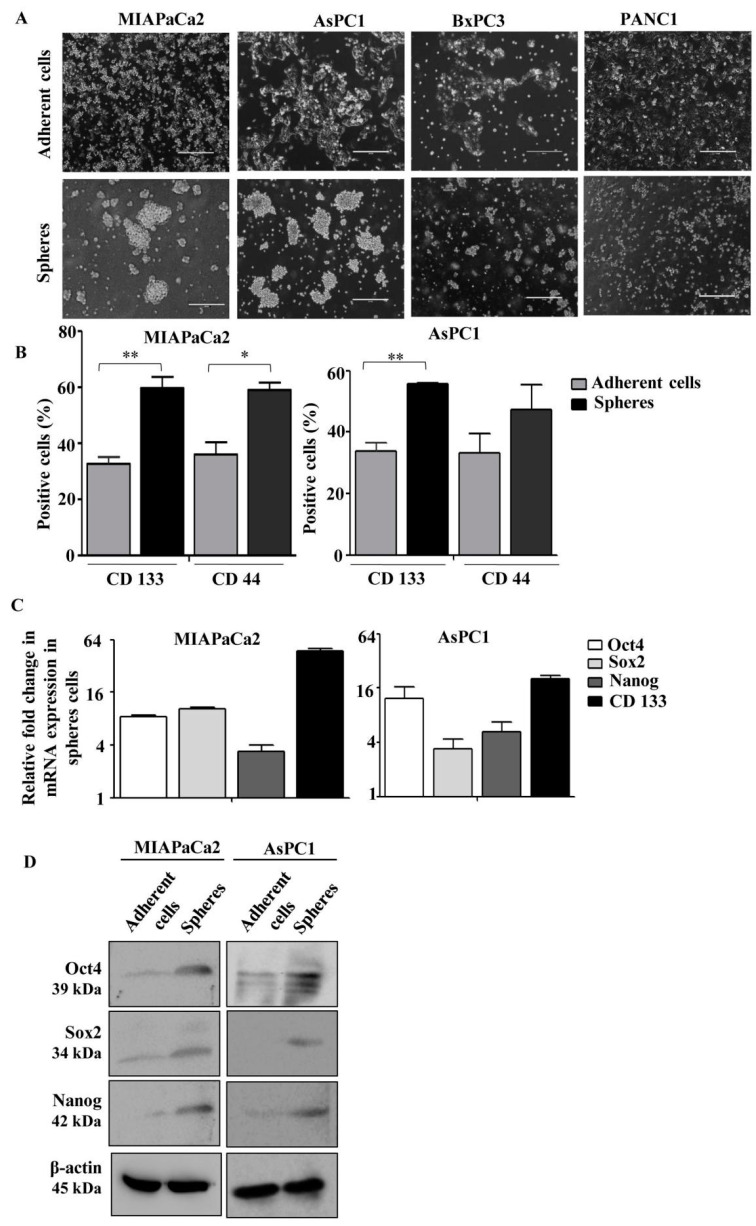
Generation of pancreatic cancer sphere-forming cells (PCS) from pancreatic cancer cell lines. (**A**) Human pancreatic cancer cell lines (MIAPaCa2, AsPC1, PANC1 and BxPC3) were cultured in non-adherent plates in stem cell-specific medium containing DMEM/F12, B-27 supplements, epidermal growth factor (EGF) and Platelet-derived growth factor (PDGF) for 3 days. Representative images show differential sphere-forming potential of pancreatic cancer cell lines. (**B**) Quantification of percentage of CD133 and CD44 positivity in adherent cancer and sphere cells from both MIAPaCa2 and AsPC1 cells. Spheres (5 × 10^5^) were collected, washed and incubated with anti-CD133-APC and anti-CD44-PE antibodies for 30 min at 4 °C in the dark. Bar graphs show higher number of CD133- and CD44-positive cells in spheres. Adherent cancer cells were processed similarly. Error bars represent the mean (±) SD; *p*-values (* *p* < 0.05; ** *p* < 0.01) calculated using Two-tailed Student’s *t*-test. (**C**) qPCR analysis of adherent vs. sphere cells showed higher expression of pluripotent stem cell markers such as OCT4, SOX2, NANOG and pancreatic CSC marker CD133 in sphere cells using specific primers as described in Supplementary Table S1. (**D**) Representative immunoblots demonstrated enhanced expression of Oct4, Sox2 and Nanog at protein level in sphere cells. Sphere cells were collected, washed and total cell lysates were prepared and further processed for Western blotting. β-actin served as a loading control. Adherent cancer cells were trypsinized and processed similarly.

Human pancreatic cancer cell lines, namely MIAPaCa2, AsPC1, PANC1 and BxPC3, having different mutation status as described before [18], were initially used for the generation of PCS in non-adherent plates in stem cell-specific medium for three days. We observed that both MIAPaCa2 and AsPC1 cells originated from the primary tumor and ascites, respectively, showed higher sphere-forming ability than the other two cell lines (Figure 1A), indicating differential stemness-like potential among these cell lines. Therefore, we selected MIAPaCa2 and AsPC1 cells for further experiments. 

Then, we characterized those spheres for CD133 and CD44 positivity through flow cytometry. All these spheres showed higher number of CD133- and CD44-positive cells than the adherent cells (Figure 1B). Moreover, these spheres exhibited enhanced expression of pluripotent stem cell markers, Oct4, Sox2 and Nanog at mRNA (Figure 1C) as well as protein levels (Figure 1D) as assessed by quantitative real-time PCR and Western blot analyses, respectively. These results indicate that spheres generated from MIAPaCa2 and AsPC1 cells possess the characteristics of pancreatic cancer stem cells.

### 3.2. Higher Sialylation and Lower Sialidase (Neu2) Expression in PCS

**Figure 2:**
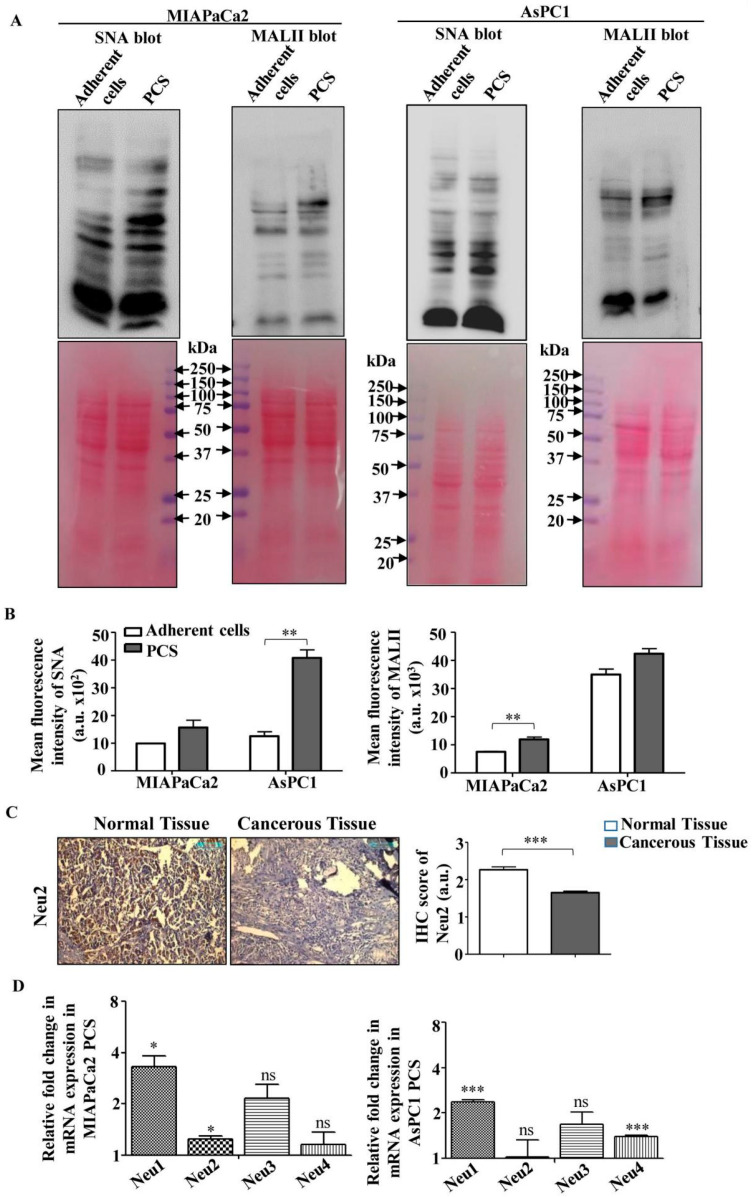
Enhanced sialylation and reduced sialidases in pancreatic cancer sphere-forming cells (PCS). (**A**) Cell lysates were prepared from both PCS and adherent cancer cells, separated electrophoretically using 10% polyacrylamide gel and further processed for Western blotting. Representative blots show enhanced *Sambucus nigra* agglutinin (SNA) and *Maackia amurensis* agglutinin (MALII) binding in PCS. Ponceau S stained blots were used as loading control. (**B**) Flow cytometry analysis revealed higher surface binding of SNA and MALII with PCS (1 × 10^5^) from both MIAPaCa2 and AsPC1 cells as described in materials and methods. PCS and adherent cells were washed and resuspended in lectin-binding buffer. Cells were incubated separately with FITC-conjugated SNA and MALII for 1 h (5.0 μg/mL at 4 °C), and FITC positivity was acquired by flow cytometry. (**C**) Immunohistological staining shows the reduced expression of Neu2 in pancreatic tumors tissues compared to adjacent normal tissues (20X magnification). Optical densitometry score showed a significant reduction in the expression level of Neu2 in tumor tissues. Scores were measured by ImageJ software. (**D**) qPCR analysis of all four sialidases, namely Neu1, Neu2, Neu3 and Neu4, in PCS vs. adherent cells was determined by using specific primers as described in Supplementary Table S1. Error bars represent the mean (±) SD; *p*-values (ns *p* > 0.05; * *p* < 0.05; ** *p* < 0.01; *** *p* < 0.001) calculated using Two-tailed Student’s *t*-test.

Next, we checked the sialylation status of these PCS derived from MIAPaCa2 and AsPC1 cells using *Sambucus nigra* agglutinin (SNA) and *Maackia amurensis* agglutinin (MALII) lectins specific for α2,6- and α2,3-linked sialic acids, respectively (Figure 2A and Figure S1A). These PCS showed a distinct higher expression of SNA-binding sialoglycoproteins than the adherent pancreatic cancer cells. However, little change was found for MALII-binding proteins in PCS. Similar trends were found in surface sialylation in these PCS as detected by flow cytometry (Figure 2B). 

The expression of sialylation is dependent on the presence of sialidases. We checked the status of Neu2 in patient tissues by immunohistochemistry in randomly selected ten pancreatic cancer patient samples. Pancreatic cancer tissues showed reduced expression of Neu2 than adjacent normal tissues as reflected in optical densitometry score (Figure 2C). Therefore, we wanted to check whether this same trend has been followed in pancreatic cancer sphere-forming cells. 

Therefore, next we checked the relative expression of sialidases in these two PCS. We have observed that the relative mRNA expression of Neu2 in PCS generated from MIAPaCa2 showed a minimal change. However, in PCS generated from AsPC1, the expression of Neu2 does not show any significant change possibly due to its very low expression (Figure 2D). We also detected higher expression of Neu1 which has α2,3-linkage specificity compared with Neu2 [15]. 

Therefore, we can conclude that the higher level of α2,6- and α2,3-linked sialic acids in PCS derived from both the cell lines might be due to the minimal expression of sialidase Neu2.

### 3.3. Neu2 Overexpression Reduces Stemness-like Properties in PCS

**Figure 3:**
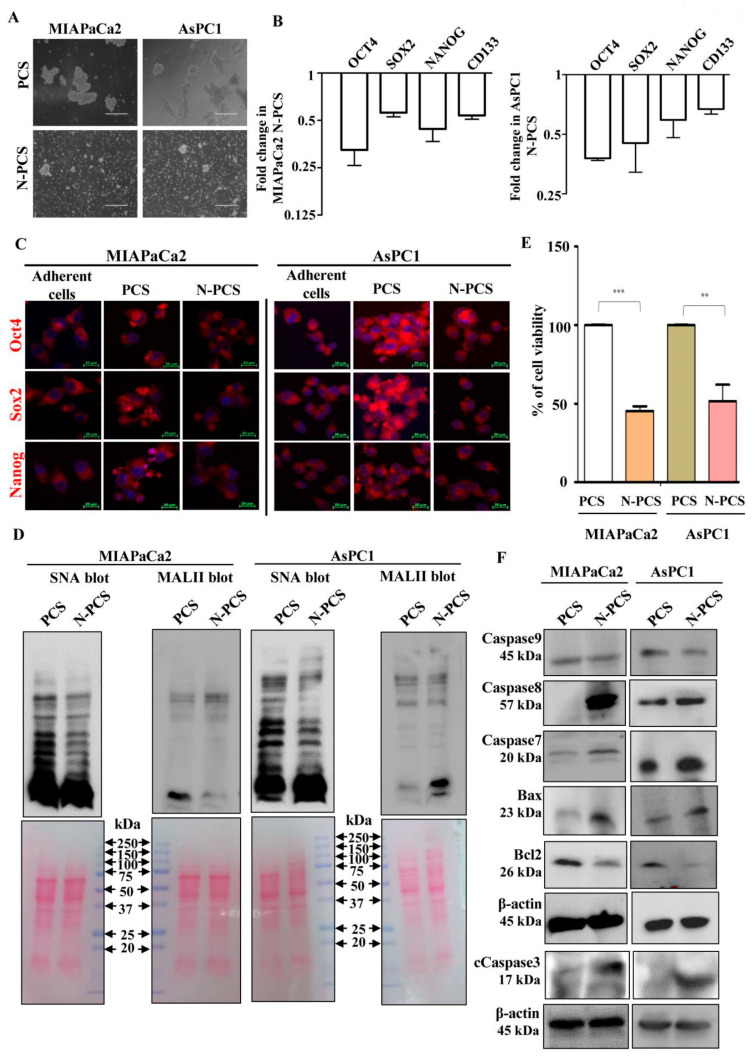
Neu2-overexpressed pancreatic cancer sphere-forming cells (N-PCS) exhibited reduced sialylation and stemness-like property. (**A**) Phase-contrast images showed reduced sphere formation of N-PCS compared to PCS generated from both MIAPaCa2 and AsPC1 cells. (**B**) Bar graph of qPCR data showed decreased expression of pluripotent stem cell markers (OCT4, SOX2, NANOG) and CD133 in N-PCS than PCS from both MIAPaCa2 and AsPC1 cells. (**C**) Confocal microscopy images showed enhanced expression of Oct4, Sox2 and Nanog in PCS compared to adherent cancer cells. Those expressions were again reduced in N-PCS. (**D**) Representative blots exhibited reduced SNA and MALII binding with N-PCS compared to PCS. Cell lysates were prepared and electrophoretically separated using 10% polyacrylamide gel and further processed for Western blotting. Ponceau S stained blots were used as loading control. (**E**) Cell viability assay showed reduced viability upon Neu2 overexpression in PCS. Error bars represent the mean (±) SD; *p*-values (** *p* < 0.01; *** *p* < 0.001) calculated using Two-tailed Student’s *t*-test. (**F**) Western blot analysis illustrated higher expression of pro-caspase proteins such as Caspase 7, 8, cleaved caspase 3 and Bax along with reduced expression of Bcl2 in N-PCS. No change of expression level was found in Caspase 9. β-actin served as a loading control.

Furthermore, we wanted to understand whether Neu2 has any role in the maintenance of stemness-like properties in pancreatic cancer sphere-forming cells. Accordingly, we have overexpressed Neu2 in PCS. After 48 h of transfection, Neu2 overexpression was confirmed in Neu2-transfected PCS (N-PCS) by Western blot analysis (Figure S1B). Neu2-overexpression resulted in a reduction of sphere-shaped morphology of these PCS (Figure 3A). N-PCS from both the cell lines also showed lower expression of pluripotent stem cell markers such as Oct4, Sox2 and Nanog, both at mRNA (Figure 3B) and protein levels (Figure S1C). CD133, a known marker for pancreatic CSCs, also decreased upon Neu2-overexpression at the mRNA level (Figure 3B). Corroborated with these data, the confocal images of N-PCS also showed reduced expression of Oct4, Sox2, and Nanog than PCS (Figure 3C). Adherent pancreatic cancer cells were used for comparison.

### 3.4. Reduction of Sialylation and Activation of Apoptosis in N-PCS

To find out whether Neu2 has any role in modulation of sialylation in PCS, we checked the overall sialylation status after Neu2 overexpression in PCS. Cell lysates from N-PCS showed downregulation of sialylated glycoproteins as there was lower binding with SNA and MALII than PCS in Western blot analysis (Figure 3D and Figure S1D). 

Next, we assessed the cell viability upon Neu2 transfection in PCS cells through MTT assay. Results showed that Neu2 overexpression caused a reduction in cell viability in PCS (Figure 3E). Furthermore, we compared a few apoptotic molecules between PCS and N-PCS. We observed higher expression of pro-apoptotic molecules at mRNA (Figure S1E) and protein (Figure 3F) levels compared to PCS. However, no such change was found in Caspase 9, a hallmark protein for intrinsic pathway-mediated apoptosis. This was corroborated with our earlier observation wherein we have reported that Neu2 overexpression activated an extrinsic pathway of apoptosis in pancreatic cancer cell lines [18].

### 3.5. Neu2 Overexpression in PCS Diminishes Hedgehog Pathway (Hh) Activity

**Figure 4:**
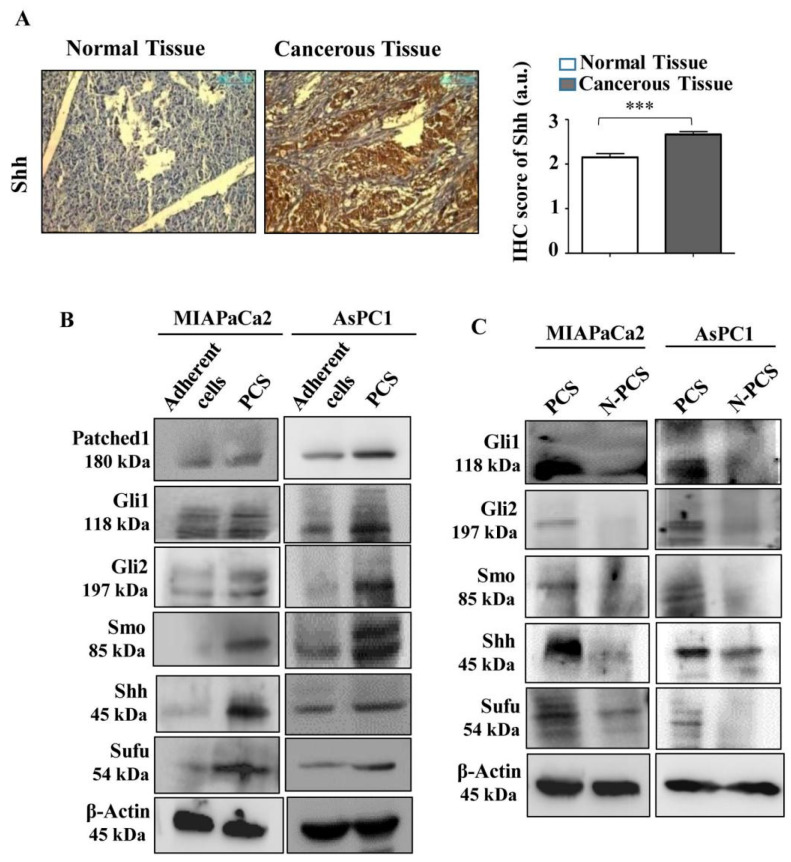
Neu2 overexpression reduced Hedgehog pathway (Hh) activity in PCS. (**A**) Immunohistological staining of pancreatic tumors and adjacent normal tissues showed the enhanced expression of Shh in cancer tissues. The tissue specimens were photographed at 20× magnification. Optical densitometry score showed significant changes in the expression level of Shh. Scores were measured using ImageJ software. Error bars represent the mean (±) SD; *p*-value (*** *p* < 0.001) calculated using Two-tailed Student’s *t*-test. (**B**) Representative immunoblots exhibited higher expression of several Hedgehog pathway proteins like Patched1, Gli1, Gli2, Smo, Shh and Sufu in PCS than in adherent cells in both MIAPaCa2 and AsPC1 cells. β-actin was used as a loading control. (**C**) Representative immunoblots demonstrated decreased expression of Gli1, Gli2, Smo, Shh, and Sufu in N-PCS than PCS from both MIAPaCa2 and AsPC1 cells. β-actin used as a loading control.

The Hh pathway is frequently hyperactivated in cancer and regulates several genes leading to cell proliferation. The sonic hedgehog protein is the main driving molecule in the Hh pathway. Therefore, we assessed the expression of Shh in patient tissues by immunohistochemistry in ten randomly selected pancreatic cancer patient samples. Representative images showed an enhancement of Shh protein in pancreatic cancer tissues as conferred by optical densitometry score compared to their adjacent normal counterparts (Figure 4A). Next, we wanted to check whether the same trend of Shh expression has been found in PCS which helps in the renewal of cancer stem cells. Therefore, we checked the expression of Hh pathway specific molecules in PCS generated from both cell lines. We found higher expression of Patched1 (PTCH1), Shh, Sufu, Smo, Gli1, Gli2 and Gli3 both at mRNA (Figure S2A) and protein levels (Figure 4B) than adherent cancer cells. Therefore, lower Neu2 expression probably helps in higher sialylation in PCS to maintain their stemness-like properties as evidenced by higher Shh expression. 

To prove our hypothesis, we checked the status of Hh pathway molecules in Neu2-overexpressed PCS (N-PCS). Corroborated with our assumption, the expression of Patched1 (PTCH1), Shh, Sufu, Smo, Gli1, Gli2 and Gli3 are downregulated in N-PCS as evidenced by lower mRNA (Figure S2B) and protein levels compared to PCS (Figure 4C). Therefore, we conclude that Neu2 plays an important role in the maintenance of stemness-like properties in PCS by modulation of the Hh pathway by regulating overall sialylation.

### 3.6. Association of Neu2 with Sonic Hedgehog (Shh) Causes Its Desialylation and Deactivation

**Figure 5:**
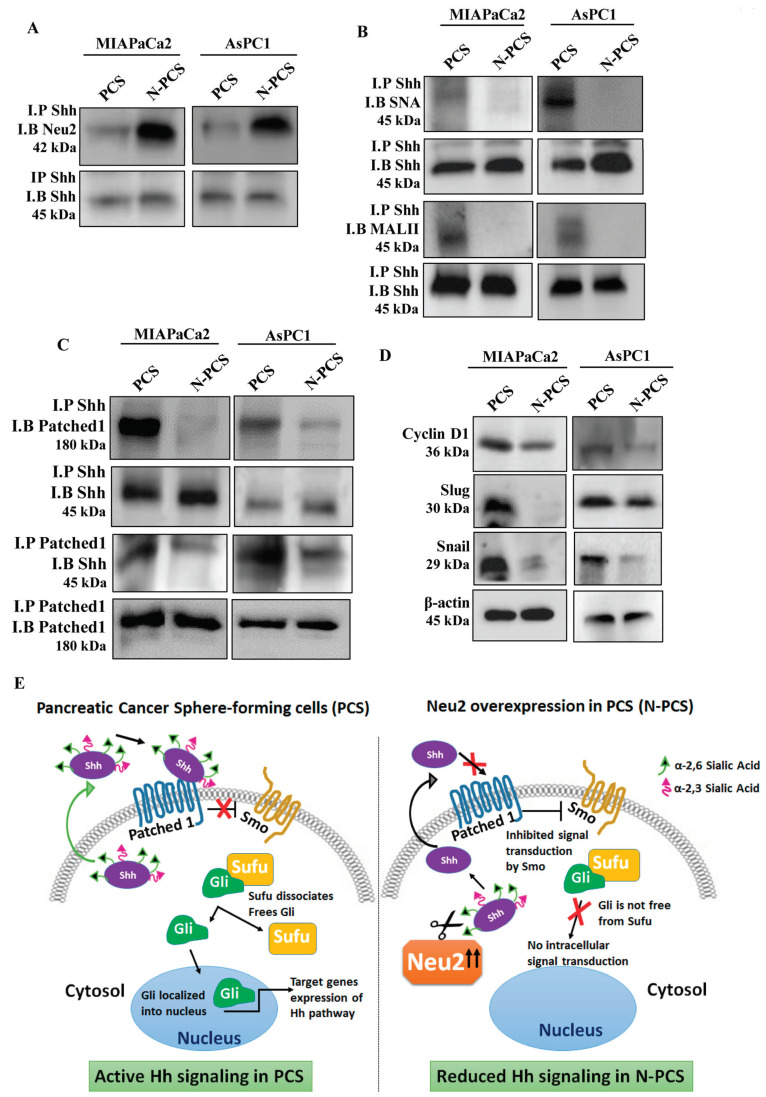
Impaired association of Shh with Patched1 due to desialylation of Shh by overexpressed Neu2. (**A**,**B**) Co-immunoprecipitation of Neu2 and Shh showed higher association in N-PCS than PCS. Cell lysate (200 μg) was incubated with the anti-Shh antibody (1:100) overnight at 4 °C. Immuno-complexes were incubated with protein A-Sepharose 4B for 3 h, resolved by SDS-PAGE, and subsequently identified using the anti-Neu2 antibodies (**A**). The immunocomplexes were also identified using the SNA and MALII, respectively, to illustrate reduced α2,6- and α2,3-linked sialylation of Shh in N-PCS (**B**). Immunoblots with anti-Shh antibody served as a loading control. (**C**) Co-immunoprecipitation showed decreased association of Patched1 and Shh in N-PCS. This was performed as described in materials and methods. Immunocomplex was identified using the anti-Patched1 and anti-Shh antibodies. Immunoblots of Shh and Patched1 served as loading controls, respectively. (**D**) Representative Western blots showed reduced expression of Hedgehog pathway target proteins such as Cyclin D1, Slug and Snail in N-PCS. β-actin was used as a loading control. (**E**) Schematic diagram highlighting reduced association of Shh with Patched1 in N-PCS due to removal of sialic acids on Shh by overexpressed-Neu2. As a result, the Hedgehog signaling cascade is downregulated by inhibiting Smo and reducing the expression of Sufu and Gli proteins.

So far, we have shown that overexpression of Neu2 modulates the Hh pathway in pancreatic cancer sphere-forming cells. Next, we wanted to address the mechanism of such modulation of the Hh pathway by Neu2. Co-immunoprecipitation of Neu2 with Shh showed higher association of Neu2 with Shh in Neu2-overexpressed PCS (N-PCS) generated from both the cancer cell lines (Figure 5A). Furthermore, co-immunoprecipitation of Shh with SNA showed less association, suggesting desialylation of highly glycosylated Shh in N-PCS compared to PCS (Figure 5B). A similar trend was observed when Shh co-immunoprecipitated with MALII. In this experiment, for the first time, we demonstrated that Shh is a sialoglycoprotein having both α2,6- and α2,3-linked sialic acids in PCS. 

Moreover, we checked the association of Shh with Patched1 which facilitates the release of Smo to further activate downstream signaling. Co-immunoprecipitation exhibited less association of Shh with Patched1 in Neu2-overexpressed PCS (N-PCS) compared to PCS (Figure 5C). Hence, it proves that the association of desialylated Shh in Neu2-overexpressed conditions was unable to bind with Patched1 which helps in downregulating the Hh pathway in N-PCS. Furthermore, Neu2 overexpression caused reduction of Hh pathway target genes such as Cyclin D1, Slug and Snail in PCS due to desialylation and deactivation of Shh molecule (Figure 5D). These events have been demonstrated pictorially in Figure 5E.

### 3.7. Neu2 Modulates Hedgehog Pathway (Hh) Activity by Downregulating mTORC2 Formation

Mammalian target of rapamycin (mTOR) is known to be associated with cancer progression as well as CSC maintenance in pancreatic cancer [28]. Rictor is the essential component to form mTOR complex 2 (mTORC2), whereas Raptor is needed to form mTORC1. We have earlier reported lower mTORC1 and mTORC2 levels in Neu2-overexpressed pancreatic cancer cells [18]. However, here we observed higher mTORC1/2 formation in PCS generated from both the cell lines as depicted by the elevated phosphorylation of mTOR both at the serine 2448 and 2481 positions, respectively, compared to adherent pancreatic cancer cells (Figure S3A). Further, we confirmed that these complex formations were facilitated by negative phosphorylation of GSK3β at Ser 9 position and positive phosphorylation of AKT at Ser 473 position in PCS (Figure S3B). 

**Figure 6:**
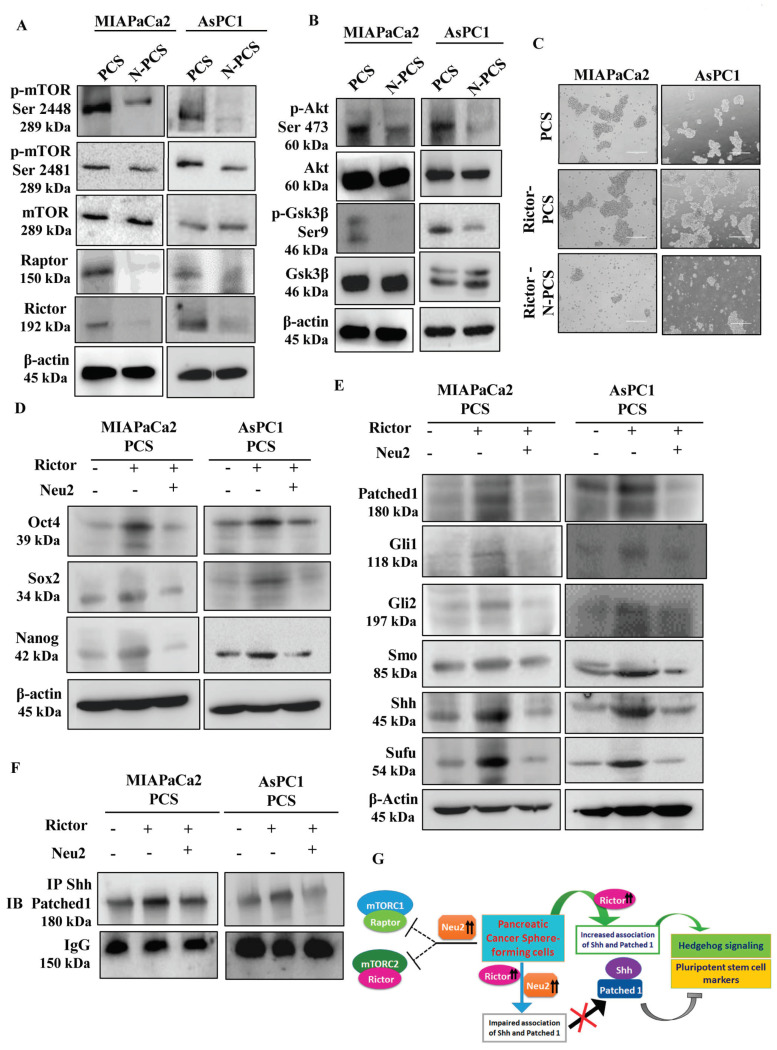
Neu2 overexpression modulates mTORC1/2 activity leading to further downregulation of Hh signaling in N-PCS. (**A**) Representative immunoblots exhibited reduced mTORC1/2 formation and decreased expression of Raptor and Rictor in N-PCS from both MIAPaCa2 and AsPC1 cells. β-actin used as a loading control. (**B**) Western blot analysis demonstrated reduced inhibitory phosphorylation of Gsk3 β at Ser 9 and decreased phosphorylation of Akt at Ser 473 in N-PCS from both the cell lines. β-actin used as a loading control. (**C**) Phase-contrast images illustrated higher sphere generation upon Rictor overexpression in PCS. Co-overexpression of Rictor and Neu2 in PCS exhibited reduction of sphere formation. (**D**,**E**) Representative immunoblots showed enhanced expression of Oct4, Sox2 and Nanog at protein level in Rictor-overexpressed PCS whereas reduced expression of these molecules was observed when both Rictor and Neu2 were co-overexpressed in PCS. β-actin used as a loading control (**D**). Similarly, increased expression of Hedgehog pathway proteins was observed in Rictor-overexpressed PCS which were reversed in Rictor and Neu2 co-overexpressed conditions. β-actin used as a loading control (**E**). (**F**) Co-immunoprecipitation of Patched1 and Shh exhibited enhanced association upon Rictor overexpression in PCS. Co-overexpression of Rictor and Neu2 in PCS exhibited reduction of association of Patched1 and Shh. Immunoblots of IgG served as a loading control. (**G**) Schematic diagram described inhibition of mTORC1 and mTORC2 formation upon Neu2 overexpression in PCS. Although Rictor overexpression promotes Hh-signalling in PCS, co-overexpression of both Rictor and Neu2 inhibit association of Shh with Patched1 leading to reduced Hh pathway and stemness-like properties.

In contrast, Neu2 overexpression caused the downregulation of mTORC1/2 formation in PCS as evidenced by the lower phosphorylation of mTOR at serine 2448 and 2481 positions and lower expression of Raptor and Rictor, respectively, in both N-PCS (Figure 6A). Additionally, Neu2-overexpressed PCS demonstrated lower negative phosphorylation of GSK3β at Ser 9 position and reduced positive phosphorylation of AKT at Ser 473 position, suggesting reduced survival of N-PCS (Figure 6B). 

Formation of mTORC2 is mainly dependent on Rictor [9,29]. Accordingly, we overexpressed Rictor in the adherent pancreatic cancer cells and placed them for sphere generation. Rictor overexpression triggered higher and larger spheres formation in both MIAPaCa2 and AsPC1 cells (Figure 6C). The higher amount of Rictor as well as the mTORC2 formation were also observed in Rictor-overexpressed PCS compared to PCS (Figure S3C). Furthermore, Rictor overexpression in PCS resulted in higher expression of pluripotent stem cell markers (Figure 6D) and Hh-pathway molecules (Figure 6E).

Next, Rictor and Neu2 were co-expressed in PCS. These cells showed less sphere formation (Figure 6C), reduced Rictor, decreased mTORC2 expression (Figure S3C) and pluripotent stem cell markers (Figure 6D) along with Hh-pathway molecules (Figure 6E).

Moreover, co-immunoprecipitation of Shh with Patched1 was greatly enhanced upon Rictor overexpression in PCS whereas co-expression of both Rictor and Neu2 reduced this association (Figure 6F). Therefore, Neu2 is capable of directly modulating the Hh pathway as well as mTORC2 formation, which are essential characteristics for the survival of PCS. These events have been demonstrated pictorially in Figure 6G.

### 3.8. Neu2 Overexpression Reduced Tumor Growth in Xenograft Model

**Figure 7:**
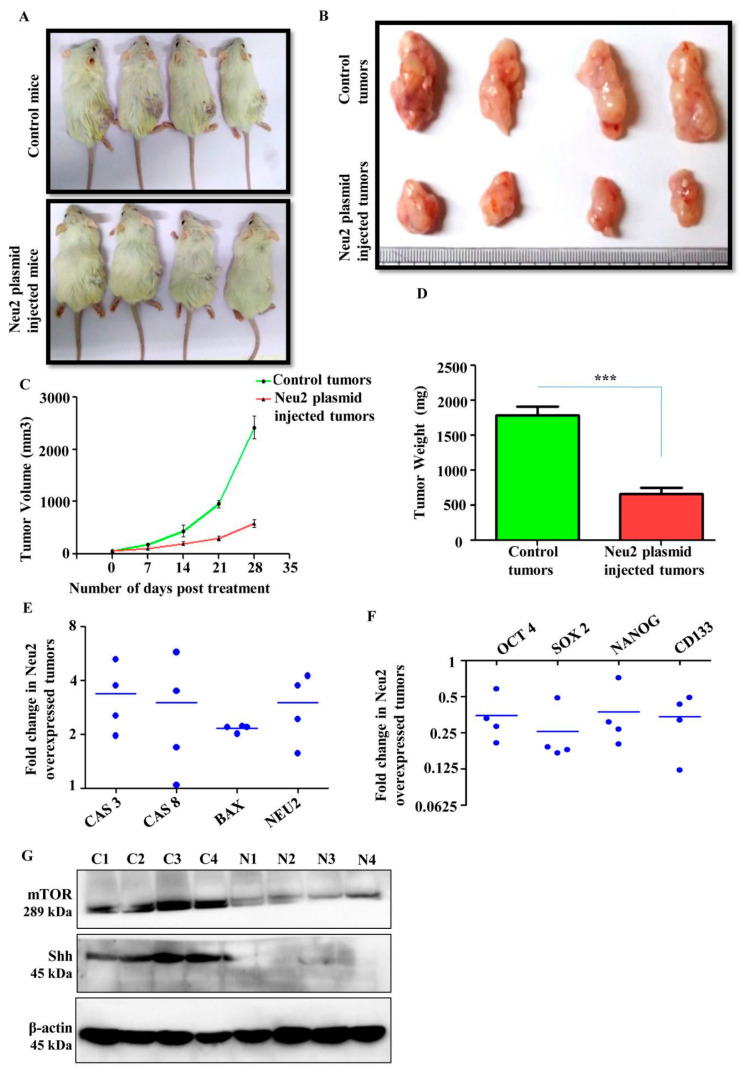
Neu2 overexpression reduced tumor growth in NOD/SCID mice. (**A**–**D**) MIAPaCa-2 (1 × 10^7^) cells were injected subcutaneously into the dorsal side of the right flanks of male NOD/SCID mice to develop tumors. Mice were either injected with vehicle control or 1.5 mg/kg body wt. Neu2-plasmid in admixture with Lipofectamine 2000 (1:2) twice a week for 3 weeks intratumorally. Pictures of tumor-bearing mice (**A**), extracted tumors (**B**), reduced tumor volumes (**C**) and reduced tumor weight were observed in Neu2-plasmid injected mice (**D**). Error bars represent the mean (±)SD; *p*-value (*** *p* < 0.001) calculated using Two-tailed Student’s *t*-test. (**E**,**F**) Tumor tissues were homogenized and single-cell suspensions were made, and subsequently total RNA was extracted using Trizol. Neu2 overexpression was confirmed at genetic level. qPCR analysis showed upregulation of pro-apoptotic genes such as CAS 3, CAS 8 and BAX in Neu2-overexpressed tumors (**E**). qPCR data showed downregulation of pancreatic cancer stem cell specific markers such as OCT4, SOX2, NANOG and CD133 in Neu2-plasmid-injected tumor samples (**F**). (**G**) Tumor tissues were homogenized and whole cell lysates were made. Representative immunoblots illustrated decreased levels of Shh and mTOR in Neu2-plasmid-injected tumor samples. β-actin used as a loading control.

For further validation of the effect of Neu2 on apoptosis, we performed an in vivo tumorigenic experiment in male NOD/SCID mice. MIAPaCa2 cells were injected subcutaneously for the generation of xenografts. After formation of palpable tumors, Neu2 plasmid was injected intratumorally twice a week for three weeks. Mice were euthanized and tumors were dissected out after 30 days. Neu2 overexpression caused a visible reduction in tumor size in NOD/SCID mice model (Figure 7A,B). There was a marked reduction in tumor volume and weight in Neu2-plasmid injected tumors as compared to the vehicle controls (Figure 7C,D). Furthermore, we analyzed the status of several genes linked to apoptosis and stemness properties through qPCR analysis of tumor tissues derived from both vehicle control and Neu2-plasmid-injected groups. We observed a significant increase in the mRNA fold change of NEU2 along with a few pro-apoptotic genes such as CASPASE 3/8, and BAX in Neu2-overexpressed tumor tissue samples as compared to vehicle control (Figure 7E). Moreover, Neu2-overexpressed tumor tissue samples showed reduction of pluripotent stem cells markers such as OCT4/SOX2/NANOG and PCS specific marker CD133 at the transcript level (Figure 7F). Furthermore, Neu2-overexpressed tumor tissue samples showed a lower expression of Shh and mTOR at protein level than vehicle control (Figure 7G). Taken together, Neu2 overexpression into pancreatic tumors generated in NOD/SCID mice led to reduction of tumor growth via modulation of mTOR/Hh axis thereby reducing stemness-like properties and inducing apoptosis.

## 4. Discussion 

Sialidases play an important role in balancing the level of sialylation in the mammalian system. Abnormal sialylation is responsible for altering many cellular signaling pathways which are responsible for cancer cell survival and metastasis [30,31]. The drug-resistant cancer cells generally possess self-renewal and differentiation properties and are considered the main source of tumor recurrence and are termed as CSCs [32]. Higher sialylation is one of the key features of CSCs which is also associated with tumor aggressiveness [33]. 

The important achievement of our study is the evidence that overexpressed Neu2 reduces the stemness-like properties of pancreatic cancer sphere-forming cells by desialylating an important molecule in the Hedgehog pathway. We have demonstrated for the first time that Shh, a key player in the Hh pathway, is a sialoglycoprotein having both α2,6- and α2,3-linked sialic acids. The association of Shh with Patched1 is the first step and is necessary for the successful initiation of downstream signaling of the Hh pathway. Our data demonstrate that sialylation of Shh is critical for this association with Patched1. Reduced association of desialylated Shh and Patched1 modulates several molecules in the Hh pathway to reduce the stemness-like property of PCS.

Overexpressed Neu2 also decreases mTORC2 formation, phosphorylation of GSK3β at Ser 9 position and phosphorylation of AKT at Ser 473 position, which also affects the Hh pathway by ubiquitinylating Gli molecule to decrease the stemness-like properties of PCS. Modulation of stemness-like properties by Neu2 was further validated in a xenograft mice model. Overall, our study conclusively demonstrates the important role of sialidase Neu2 in reduced survival of pancreatic cancer sphere-forming cells which exhibited pancreatic cancer stem cell-like properties by modulating both Shh and mTORC2 axis.

The expression of cytosolic sialidase (Neu2) is much less both in pancreatic cancer cell lines and patient tissues. Due to such reduced expression of Neu2, earlier we have demonstrated higher sialylation both in MIAPaCa2 and AsPC1 having K-RAS-activating and p53-inactivating mutations indicating more aggressiveness of these two cell lines [18].

Here we observed that Neu2 expression was also low in PCS, which is possibly responsible for higher sialylation specially α2,6- and α2,3-linked sialic acids, which are substrates for this sialidase Neu2. Sialylation plays an important role in regulating pluripotency and differentiation in stem cells and is also involved in crucial cell fate decisions [34,35]. Hence, the upregulation of this sialidase in cancer stem cells which cleave sialic acids from glycoproteins and oligosaccharides is expected to deregulate the stemness-like property. As expected, we observed reduced sphere formation capacity, as well as higher sialylation status in the PCS, generated both from MIAPaCa2 and AsPC1 compared to adherent pancreatic cancer cell lines where Neu2 was overexpressed. Neu2 overexpression also caused a reduction in several stem cell-specific molecules such as Oct4, Sox2 and Nanog in these N-PCS. Furthermore, this upregulation of sialidase caused a reduction in the sialoglycoprotein profiles.

Hence, this detrimental effect in the modification of the sialylation profile by Neu2 overexpression directed these N-PCS towards apoptosis as exhibited by an increase in the several pro-apoptotic and decrease in the anti-apoptotic molecules. This was corroborated with our earlier report that Neu2 overexpression guides upregulation of Fas, FasL and FADD, leading to activation of caspase8 and Bid cleavage, thus inducing extrinsic pathway-mediated apoptosis not only in pancreatic [18] but also in ovarian cancer cells (personal communication). Therefore, it may be hypothesized that cytosolic sialidase Neu2, in general, preferentially aims at the extrinsic pathway-mediated apoptosis through targeting several membrane-bound molecules not only in different cancer cells but also in pancreatic cancer sphere-forming cells. Neu2 also possesses a role in cell differentiation [36] with its overexpression leading leukemic cells towards apoptosis [16].

The Hh pathway is an important signal transduction pathway for the maintenance and proliferation of cancer stem cells [3,37]. Spheres generated from MIAPaCa2 and AsPC1 cell lines (PCS) also showed higher activation of the Hh pathway than the adherent cancer cells. Lower expression status of Neu2 and higher expression of Shh were found in patient tissues compared to their normal counterpart. These observations encompassed the prelude of our study to assess the effects of Neu2 on the Hh pathway in PCS.

Neu2 overexpression caused downregulation in the Hh signaling pathway in PCS. Shh is the main driving molecule for activation of this pathway [7]. We had observed enhanced association of Shh with Neu2 in N-PCS. Such association leads to desialylation of Shh which was confirmed by reduced SNA and MALII binding, suggesting a presence of linkage-specific sialic acids on Shh. Although glycosylation in Shh is only reported in bladder cancer [7], this is the first report for the presence of sialic acids on Shh in PCS. 

Both Shh and Patched1 are upstream molecules for activation of this Hh pathway. Association of Shh and Patched1 is required to release Smo molecule needed to switch on this pathway [4]. This association is drastically hindered when sialic acids were removed from Shh due to Neu2 overexpression in PCS which possibly responsible for downregulation of the Hh pathway and ultimately reduced the stemness-like property of these cells as reflected by decreased expression of Oct4, Sox2 and Nanog. 

We had previously reported that Neu2 reduces mTORC2 formation in pancreatic cancer cells [18]. We also demonstrated crosstalk between mTORC2 and the Hh pathway in glioblastoma multiforme [9]. Deregulation of mTORC2 and Hh signaling help each other in increasing stemness-like properties in glioblastoma cell lines [9]. So, our next obvious question is to address the missing link between mTORC2 and Hh pathway within PCS in the context of Neu2 overexpression.

Reduced mTORC2 formation in Neu2-overexpressed PCS diminished its activity as evidenced by reduced negative phosphorylation of GSK3β at Ser 9 position and decreased phosphorylation of AKT at Ser 473 position in N-PCS. This phenomenon also affects the Hh pathway by ubiquitinylating Gli molecule and ultimately causing reduction of the survival of N-PCS as revealed by reduced expression of Oct4, Sox2 and Nanog.

Formation of the mTORC2 protein complex is highly dependent on the association of Rictor with mTOR [9,24]. As expected, Neu2-overexpressed PCS also demonstrated downregulation of both Rictor and mTORC2. Interestingly, PCS with overexpression of both Rictor and Neu2 exhibited reduced mTORC2 formation and Hh pathway activity as well as decreased cancer stem cell-specific markers, suggesting a dominant role of Neu2 over Rictor and emphasizing the involvement of sialic acids in the mTORC2-Hh pathway also.

Therefore, crosstalk between Rictor, mTORC2 and Hh pathway in the context of Neu2 overexpression in pancreatic cancer sphere-forming cells have highlighted their effect in maintaining the stemness-like property. This missing link between mTORC2 and Hh pathway with respect to the minute balance of sialidases and sialic acids would pave the way for the management of cancer stem cells in pancreatic cancer. 

Furthermore, in vivo model in NOD/SCID mice also confirmed lower mTOR activity, Shh expression, reduced stemness markers and upregulation of apoptotic molecules in Neu2-overexpressed tumor tissue samples. 

**Figure 8:**
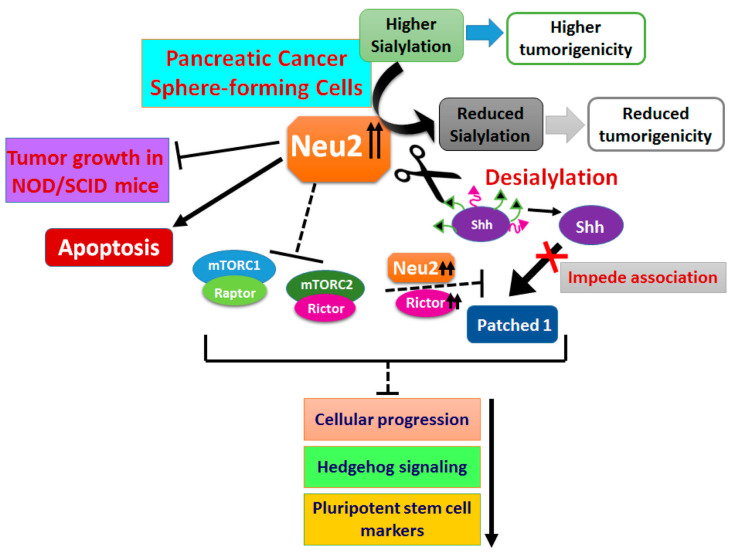
Schematic diagram of mechanism of action of Neu2. PCS possess higher sialylation and tumorigenic properties than adherent cancer cells. Overexpression of Neu2 in PCS (N-PCS) leads to a global change in sialylation leading to reduced tumorigenicity. Neu2 overexpression in PCS caused desialylation of Shh leading to impaired association with Patched1 thereby reducing Hh signaling and stemness-like properties. Overexpressed Neu2 inhibit mTORC1/2 activity thereby inhibiting cellular proliferation and subsequently inducing apoptosis as corroborated in NOD/SCID mice model.

Thus, in a nutshell, Neu2 overexpression inhibits the Hh pathway through Shh desialylation and also by downregulating the mTORC2–GSK3β axis. This clearly demonstrates that overexpressed Neu2 through desialylation of Shh alone or possibly with other Hh-pathway molecules plays a significant role in controlling the stemness-like property of N-PCS. Thus, we have established the role of Neu2 in the mTORC2–Hh pathway axis in Neu2-overexpressed PCS. More importantly, this event shoved Neu2-overexpressed PCS toward apoptosis, suggesting Neu2 as a promising target for the initiation of cell death in pancreatic cancer sphere-forming cells. All these events have been demonstrated pictorially in Figure 8. Altogether, we also observed that Neu2, in general, preferred the extrinsic pathway-mediated apoptosis by targeting several membrane-bound molecules both in adherent as well as sphere-forming cells.

## Supplementary Materials

The following are available online at https://www.mdpi.com/2073-4409/9/6/1512/s1, Supplementary Figure S1, Supplementary Figure S2, Supplementary Figure S3, Supplementary Table S1: List of Primers.
