# Supplementary material for: Desialylation of Sonic-Hedgehog by Neu2 Inhibits Its Association with Patched1 Reducing Stemness-Like Properties in Pancreatic Cancer Sphere-forming Cells
Source: Cells. 2020 Jun 21;9(6):1512. doi: 10.3390/cells9061512 (PMC7349614; doi:10.3390/cells9061512)
Supplement: Supplementary file 1 [file cells-09-01512-s001.pdf]

S1

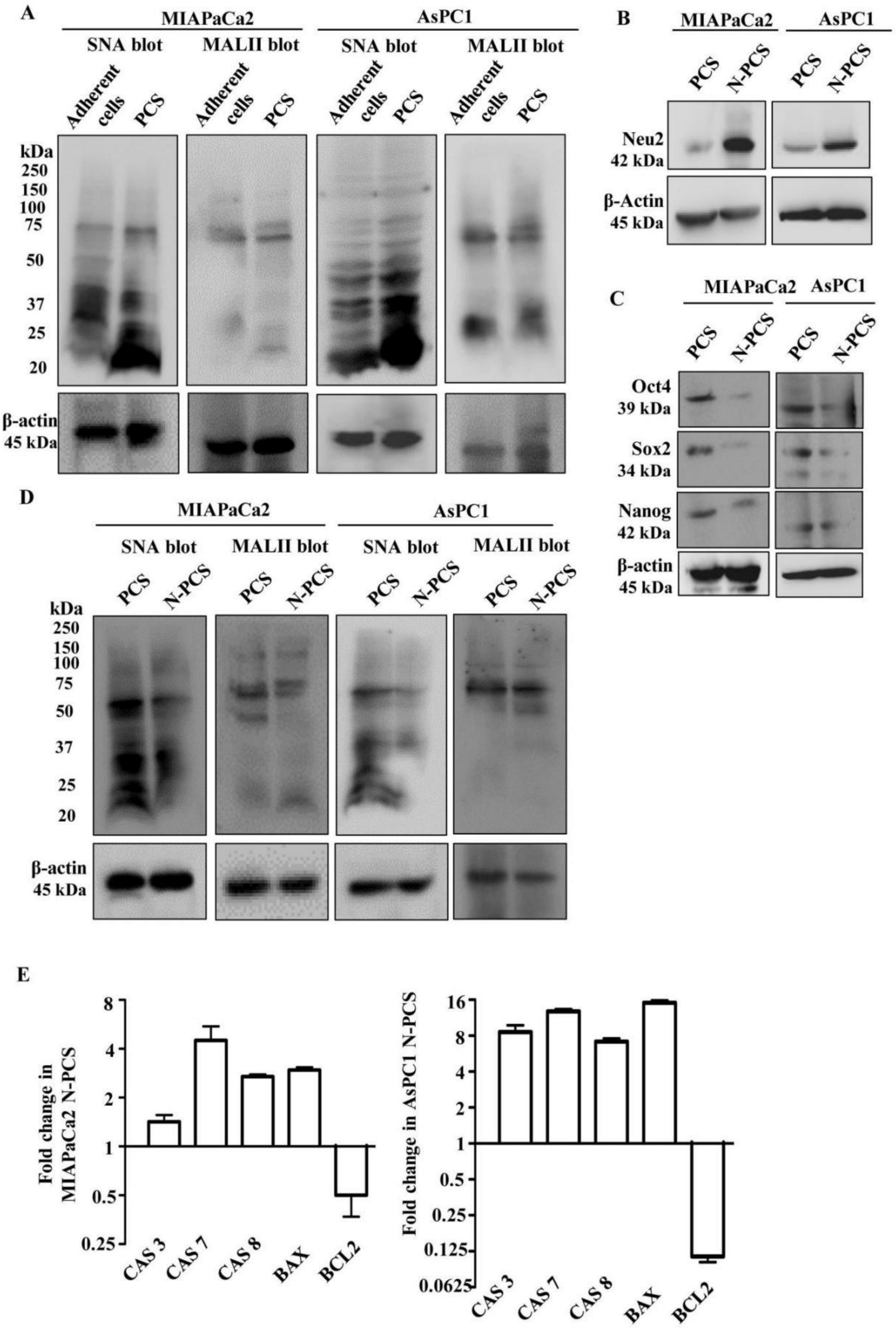

Supplementary Figure S1: (**A**) Western blot analysis of whole cell lysate from adherent and PCS. Representative blots show enhanced SNA and MALII binding in PCS. β-actin used as a loading control. (**B**) Representative immunoblots exhibited higher expression of Neu2 upon overexpression in PCS from both MIAPaCa2 and AsPC1 cells. β-actin used as a loading control. (**C**) Western blot analysis demonstrated reduced expression of pluripotent stem cell markers Oct4, Sox2 and Nanog in Neu2-overexpressed PCS. β-actin used as a loading control. (**D**) Western blot analysis of whole-cell lysate from PCS and N-PCS. Representative blots show decreased SNA and MALII binding in

N-PCS. β-actin used as a loading control. **(E)** qPCR analysis shows higher expression of pro-apoptotic genes such as CAS 3, 7, 8 and BAX and reduced expression of anti-apoptotic BCL2 in Neu2-overexpressed PCS from both MIAPaCa2 and AsPC1 cells.

S2

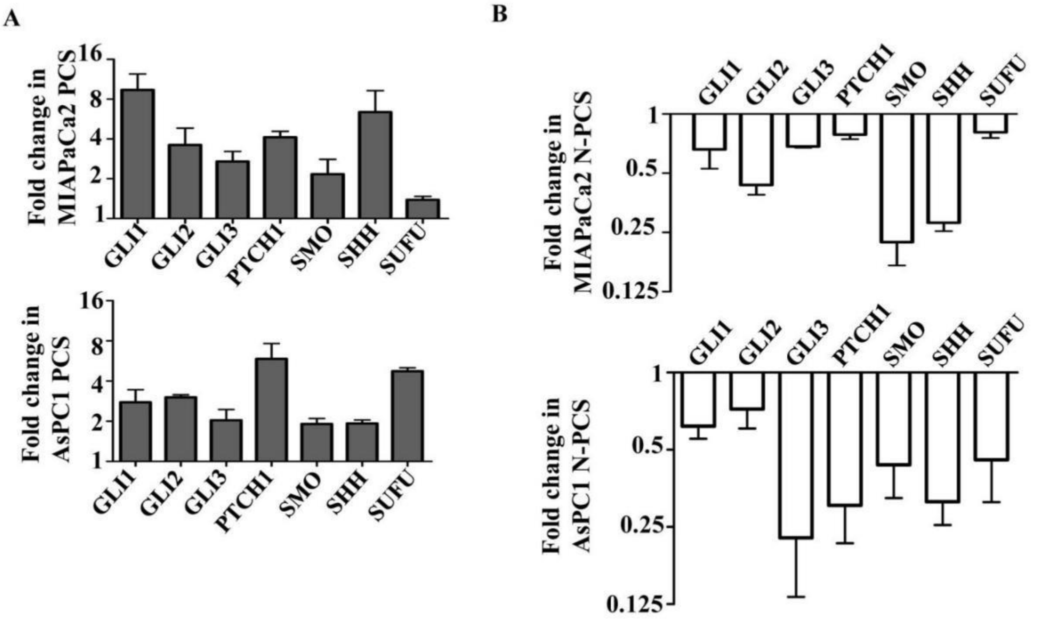

Supplementary Figure S2: (**A**) qPCR data represent an enhanced expression of Hedgehog pathway genes such as PTCH1, GLI1, GLI2, GLI3, SMO, SHH and SUFU in pancreatic cancer sphere-forming cells generated from both MIAPaCa2 and AsPC1 cells. (**B**) qPCR analysis showed reduced expression of Hedgehog pathway genes such as PTCH1, GLI1, GLI2, GLI3, SHH, SUFU and SMO in Neu2-overexpressed PCS generated from both MIAPaCa2 and AsPC1 cells.

S3

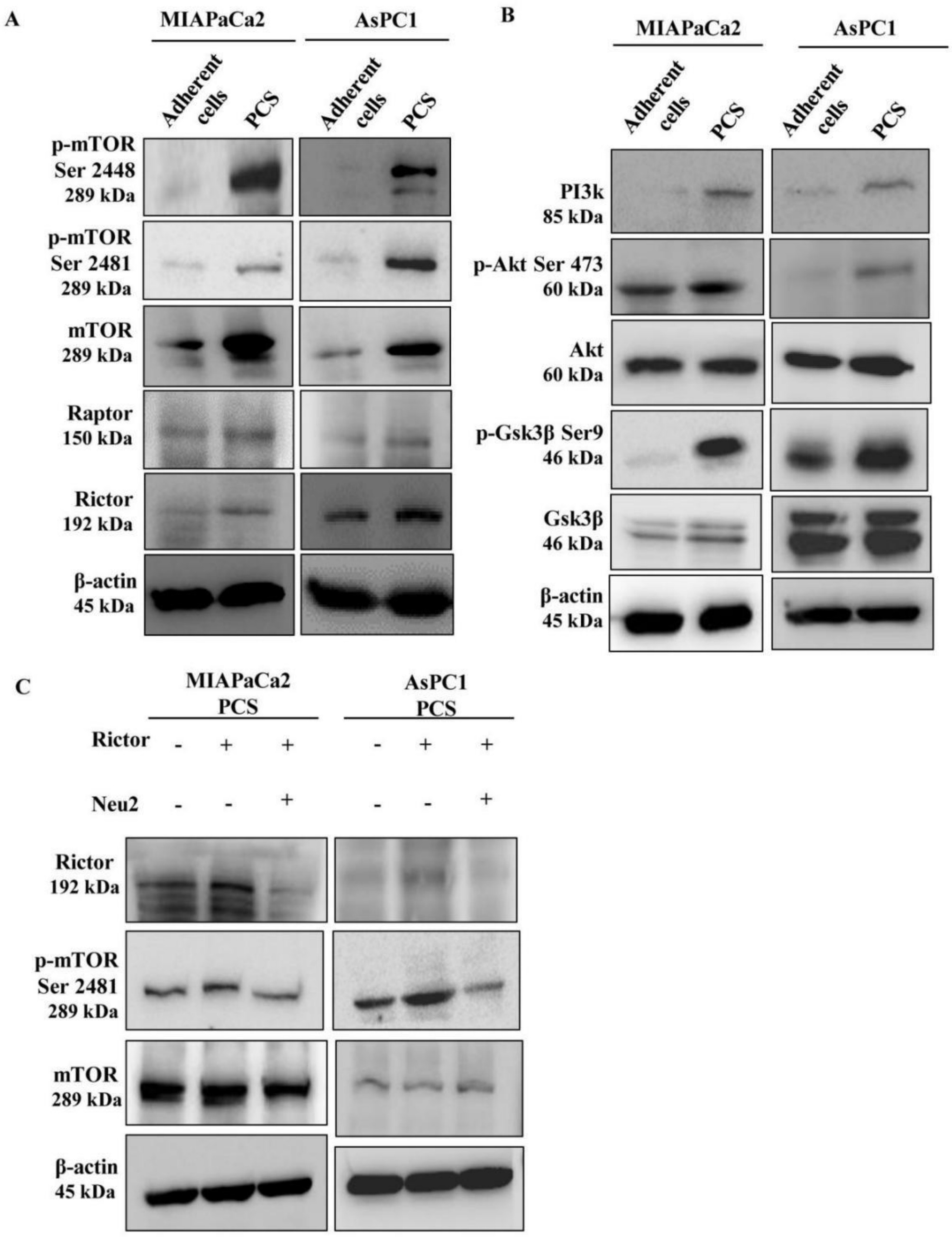

Supplementary Figure S3.: (**A**) Representative immunoblots exhibited enhanced mTORC1/2 formation and higher expression of Rictor and Raptor in PCS from both cell lines. β-actin used as a loading control. (**B**) Western blots analysis show inhibition of Gsk3β and activation of Akt in PCS from both cell lines. β-actin used as a loading control. (**C**) Representative immunoblots exhibited enhanced mTORC2 formation and Rictor expression at protein levels in Rictor-overexpressed PCS whereas reduced expression of these molecules was found in Rictor and Neu2 co-overexpressed PCS.

Supplementary Table 1: List of Primers.

NAME	FORWARD PRIMER (5′-3′)	REVERSE PRIMER (5′-3′)
OCT 4	GAGGAGTCCCAGGACATCAA	ACACTCGGACCACATCCTTC
SOX 2	AGAACCCCAAGATGCACAAC	ATGTAGGTCTGCGAGCTGGT
NANOG	ACCAGACCCAGAACATCCAG	CTCGTCGATTAGGCTCCAAC
CD133	TTGTGGCAAATCACCAGGTA	TCAGATCTGTGAACGCCTTG
GLI1	GTGCAAGTCAAGCCAGAACA	ATAGGGGCCTGACTGGAGAT
GLI2	TTTATGGGCATCCTCTCTGG	AAGGCTGGAAAGCACTGTGT
GLI 3	TCCCAGCGCTTTCTACATCT	CTTTGTCGTGGACCCATTCT
PTCH1	TCCCAGCGCTTTCTACATCT	CTTTGTCGTGGACCCATTCT
SHH	TACTCGCAGCTGCTCTACCA	TGTCTTTTTGCTTTGCGTTG
SMO	CAACCTGTTTGCCATGTTTG	TTTGGCTCATCGTCACTCTG
Neu1	GGAGCAAGGATGATGGTGTT	CATGATCATCGCTGAGGAGA
Neu2	CCTCTTCTTCATTGCCATCC	GTCGTGAAGCTGCAAACAAT
Neu3	CAGATTGTGTCAGGCAGGAA	AGCTGGAAGCAAAAGAACCA
Neu4	CACCGTCTTCCTCTTCTTCA	GGCAGATCTTGCCAAAACA
Caspase 8	AGACTGATTCAGAGGAGCAA	GTGACTGGATGTACCAGGTT
Caspase 7	GATTTGACAGCCCACTTTAG	TTCCACTGGGATCTTGTATC
Caspase 3	AGGAGCAGTTTTGTTTGTGT	CAGGCCTGAATAATGAAAAG
Bax	TCACTGAAGCGACTGATGT	GAGGAAAAACACAGTCCAAG
Bcl2	ATTGTGGCCTTCTTTGAGT	TACAGTTCCACAAAGGCATC
18s rRNA	GCTCATTAAATCAGTTATGG	ACTACCATCGAAAGTTGATA
